# Oropharyngeal stenosis in patient with oral submucous fibrosis: a case report with 8-year follow-up

**DOI:** 10.1186/s12903-024-04467-4

**Published:** 2024-06-13

**Authors:** Maged Ali Al-Aroomi, Jie Chen, Canhua Jiang

**Affiliations:** Department of Oral and Maxillofacial Surgery, Center of Stomatology, Xiangya Hospital, Central South University, Changsha, Hunan province 410008 China

**Keywords:** Oral submucous fibrosis, Oropharyngeal stenosis, Betel quid, Fibrosis

## Abstract

Oral submucous fibrosis (OSF) is a chronic, progressive condition affecting the oral mucosa associated with areca nut consumption. It leads to restricted tongue movement, loss of papillae, blanching and stiffening of the mucosa, difficulty in opening the mouth, and challenges in eating due to inflammation and fibrosis. This report presents a rare case of oropharyngeal stenosis secondary to OSF in a 43-year-old male with a history of chewing betel nut. A surgical procedure similar to Uvulopalatopharyngoplasty was performed to excise the submucous oropharyngeal stenosis and to reconstruct the uvula, palatoglossal arch, and palatopharyngeal arch. At 8 years postoperatively, the patient exhibited a normal mouth opening and oropharyngeal aperture.

## Introduction

Oral submucous fibrosis (OSF) is a progressively worsening, chronic inflammatory condition mainly impacting the oral mucosal epithelium and the underlying lamina propria. This condition leads to various levels of fibrosis throughout the oral mucosa, including the area surrounding and over the uvula. Previous studies have indicated a complex, multifaceted cause with a significant link to the consumption of areca nuts [1, 2]. Patients with OSF exhibit symptoms like restricted tongue movement, loss of tongue papillae, blanching and leathery texture of mucosa, difficulty opening the mouth (trismus), difficulty eating, and the development of vesicle due to inflammation near the epithelium, followed by fibroelastic changes in the lamina propria and epithelial atrophy [3]. With a malignant transformation rate ranging from 3 to 19%, early detection and treatment of OSF becomes crucial in pediatric patients and epithelial atrophy [4, 5]. 

Oropharyngeal stenosis, an unusual condition, is rarely mentioned in contemporary textbooks. Before the introduction of antibiotics in 1940, most pharyngeal stenosis cases were attributed to syphilis. Today, although extremely rare, it mainly occurs in children after procedures like adenotonsillectomies [6–8]. Despite extensive research, there have been no documented cases of oropharyngeal stenosis resulting from chewing betel quid.

## Case-report

A 43-year-old male was referred to the Department of Oral and Maxillofacial Surgery at Xiangya Hospital, Central South University, for assessment and management of progressive trismus with the main complaint of difficulty in eating, a burning sensation when consuming spicy foods, and limited mouth opening (10 mm interincisal distance). He noted that these symptoms had been progressively worsening over the last two years but became more severe recently (only liquid food might pass with relative ease). Additionally, he experienced a change in his voice and restricted tongue movement over the past three months. The patient’s history included chewing betel nuts over 15 years, but he denied any other significant medical or family issues.

Thus, based on our experience with such cases, combined with the patient’s history and clinical signs, OSF was initially diagnosed. The patient underwent treatment with weekly intralesional injections of salvianolic acid B combined with triamcinolone acetonide. A month later, during the examination, whitening of the entire oral mucosa was observed, and the patient was advised to continue with the intralesional injections. Three months later, a noticeable improvement in mouth opening was observed, approaching normal mouth opening (35 mm interincisal distance). Seeking further treatment, the patient came to our hospital, was admitted, and diagnosed with OSF.

**Fig. 1:**
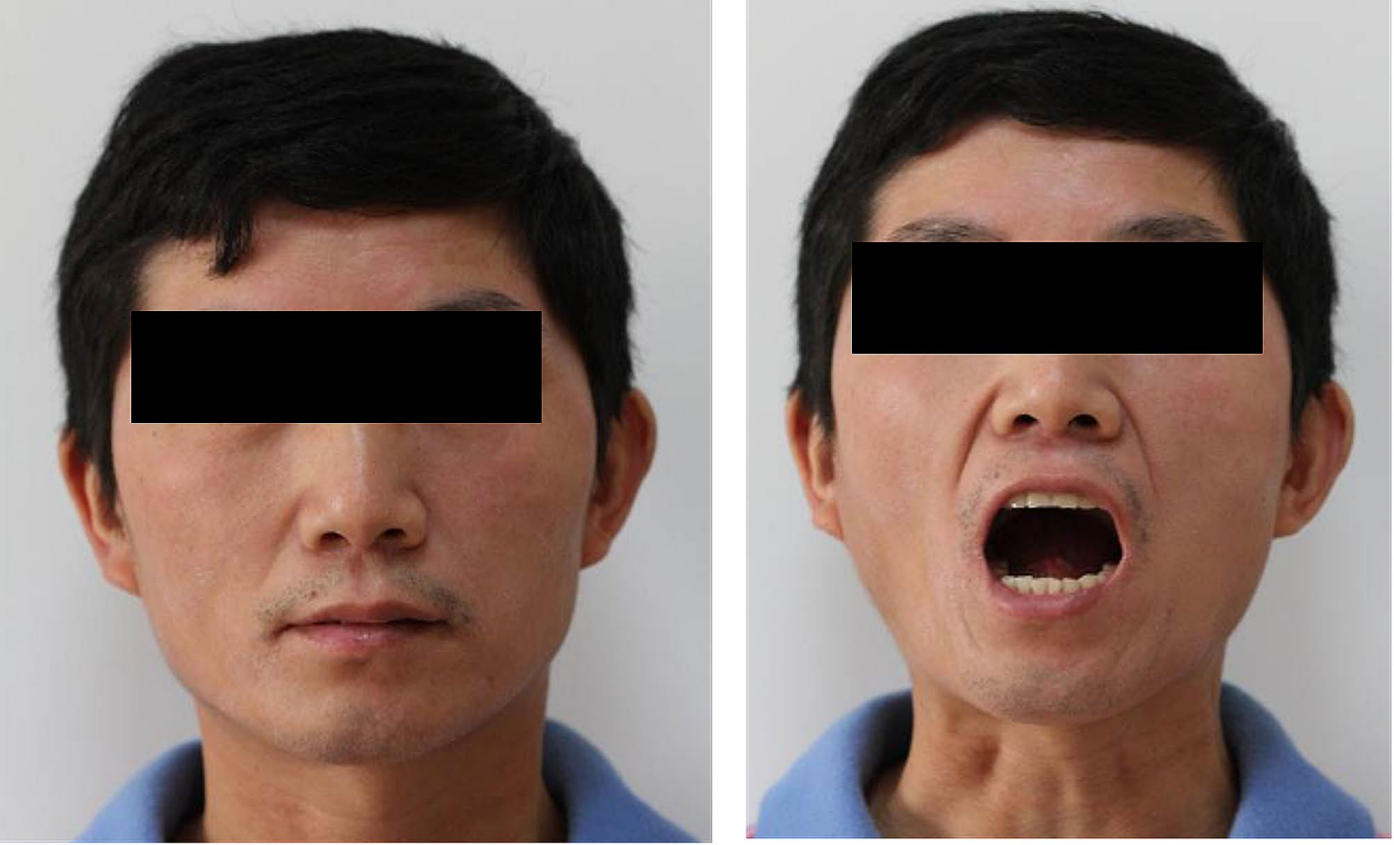
Extraoral photos of a 43-year-old male showing facial symmetry and normal mouth opening

**Fig. 2:**
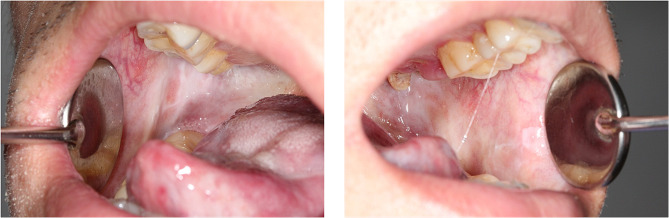
Photos showing blanching of the bilateral buccal mucosa, characterized by a hardened texture and star-like striations

**Fig. 3:**
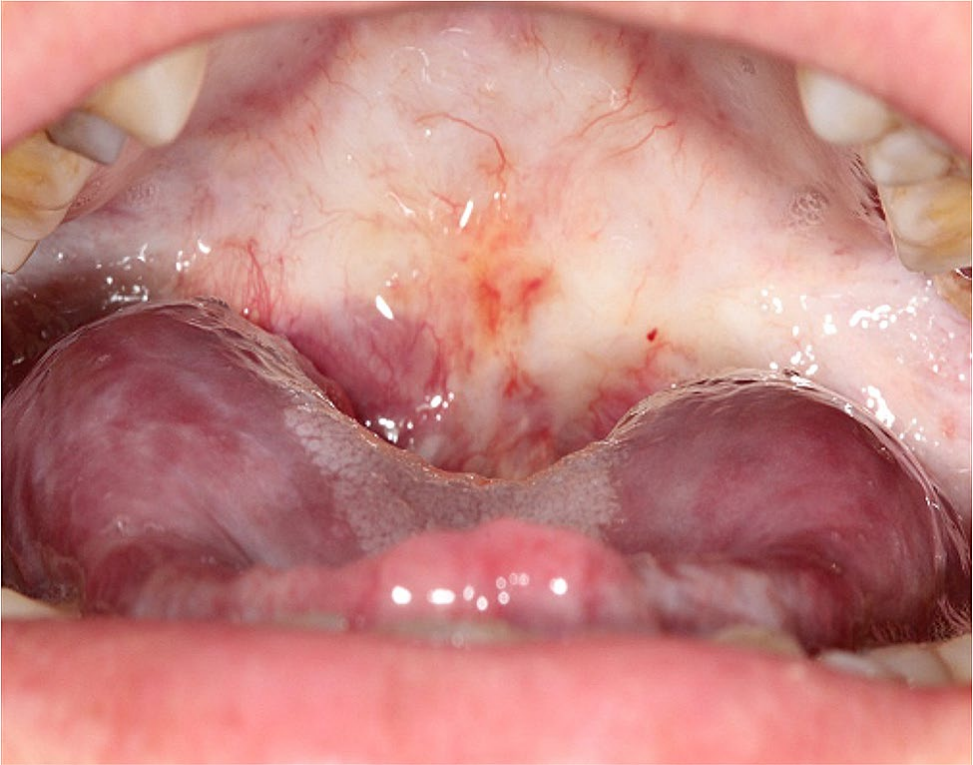
The tongue is soft, well-defined, and has a dark purple hue, but with restricted movement

Clinical examination indicates symmetry face and normal mouth opening (Fig. 1). Intraoral examination revealed blanching of the throat mucosa, bilateral buccal mucosa, and palate, all presenting a hardened texture and star-like striations, along with an absent uvula (Fig. 2). There is an enlargement at the tongue’s base and a notable adhesion of the tongue’s tip to the floor of the mouth, which restricts its mobility. The tongue itself is soft, well-defined, and has a dark purple hue (Fig. 3). No signs of tenderness or numbness are detected, and no further obvious abnormalities are noted.

**Fig. 4:**
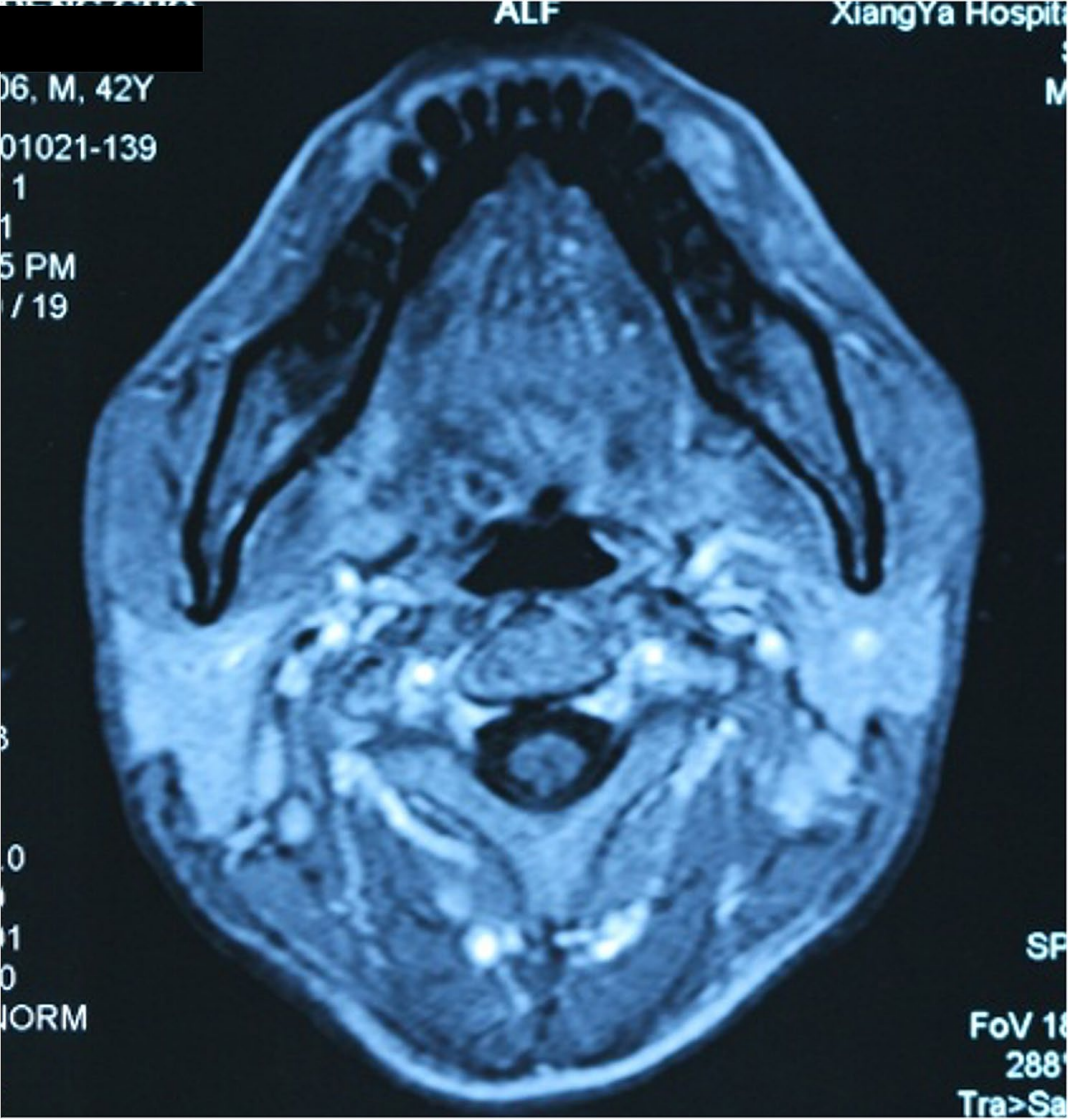
An MRI image shows the thickening of the nasopharyngeal mucosa

Nasopharyngoscopy examination showed elevation of the mucosa at the base of the tongue and the absence of neoplasms in the hypopharynx. Magnetic resonance imaging (MRI) revealed thickening of the nasopharyngeal mucosa and abnormal signals on the right side at the base of the tongue, with unclear cause (Fig. 4).

**Fig. 5:**
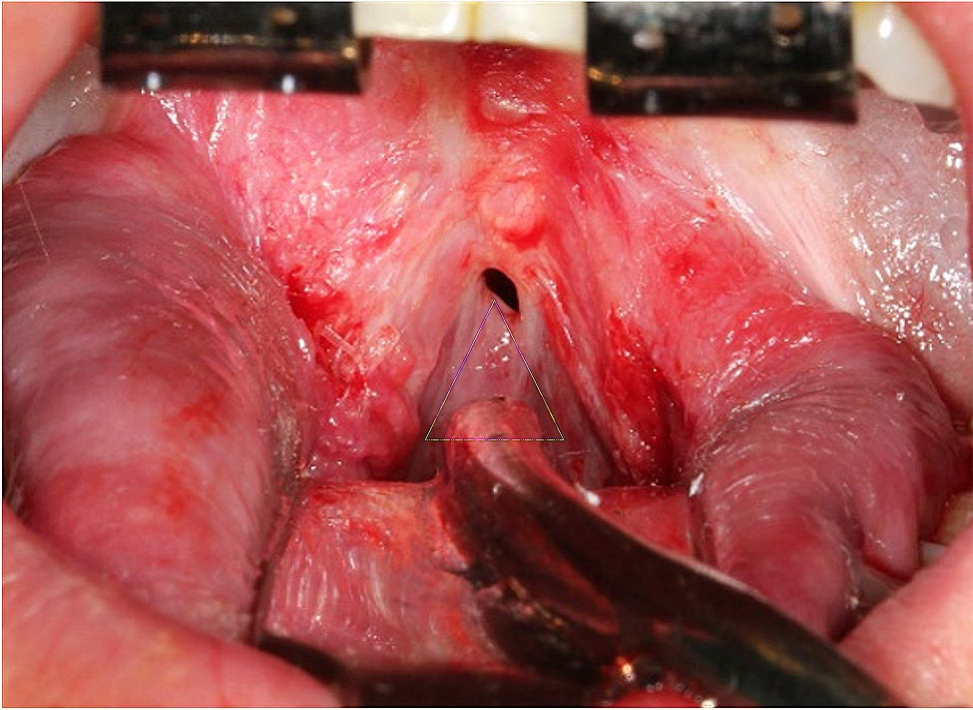
An intraoperative photo showing severe circumferential constriction of the oropharynx, creating a narrow, circular oropharynx opening measuring 0.5 cm in diameter

**Fig. 6:**
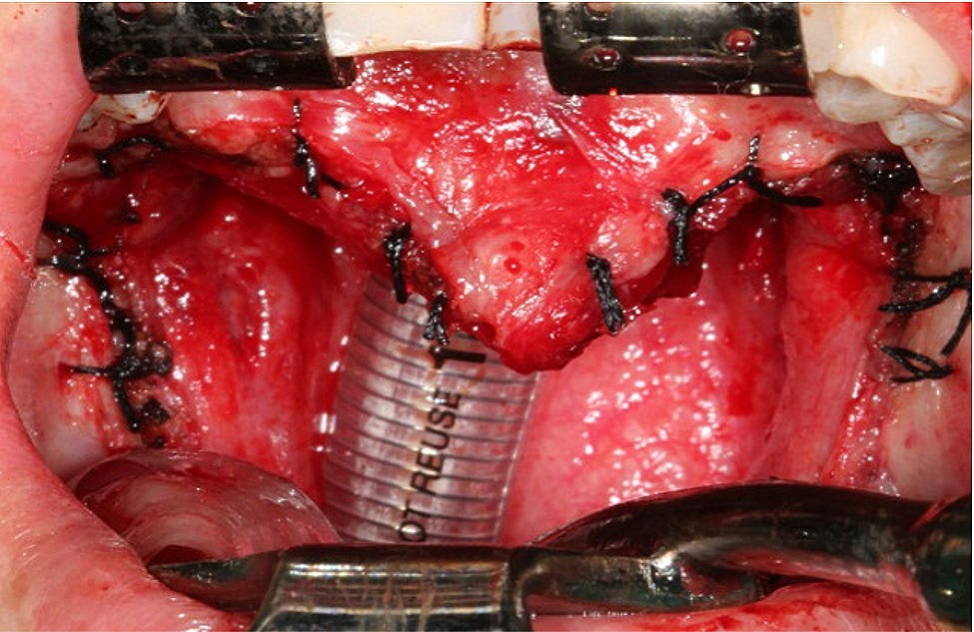
After reconstruction of the uvula, palatoglossal arch, and palatopharyngeal arch, the surface layer was sutured

**Fig. 7:**
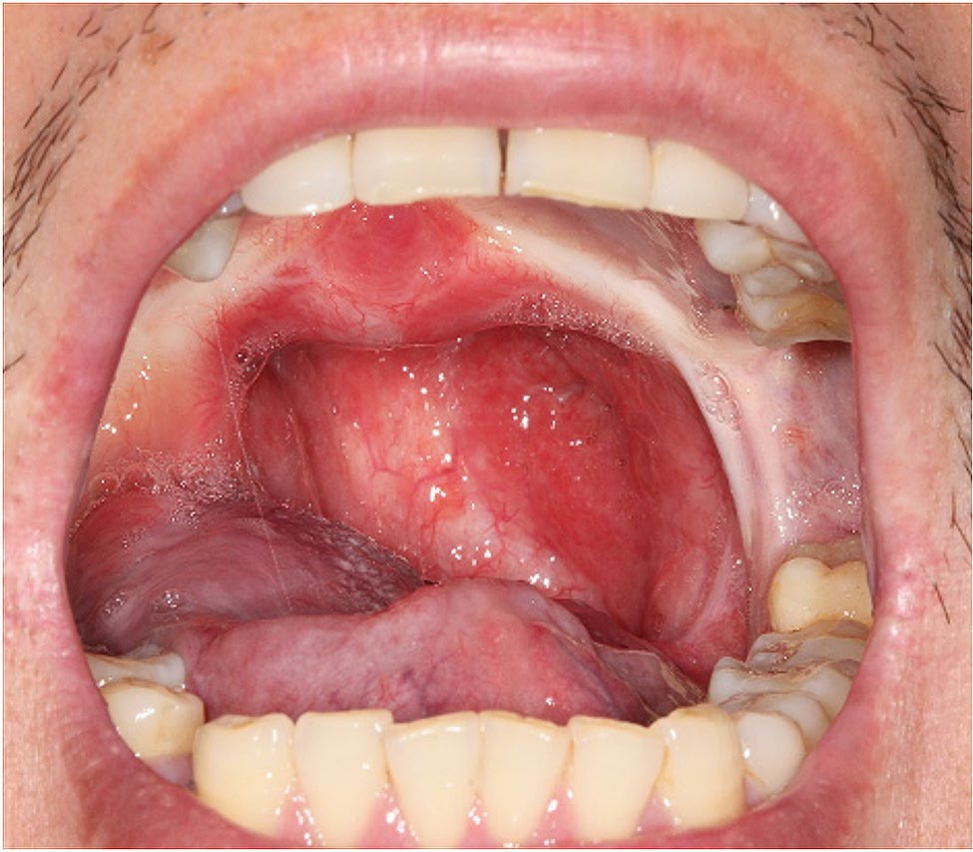
A follow-up photo 8 years postoperatively shows the patient with a normal mouth opening and oropharyngeal aperture

A surgical intervention was performed under general anesthesia to resect the submucous oropharyngeal stenosis. During surgery, it was observed that the mucosa connected the anterior pillars, the base of the tongue, and the soft palate, creating a narrow, circular oropharyngeal opening of 0.5 cm in diameter (Fig. 5). The patient underwent surgery similar to Uvulopalatopharyngoplasty involved the following steps: (1) A marginal incision was made along the edge of the oropharyngeal aperture. (2) The oral and nasal layers were separated. (3) Oblique incisions through the oral mucosa were made, originating from the anterior and posterior edges of the aperture and extending laterally on both the right and left sides. (4) Excision of the submucous oropharyngeal stenosis was performed, as highlighted in yellow lines (Fig. 5). (5) The muscles, particularly the palatoglossus, palatopharyngeus, and uvula muscles, were dissected from the nasal layer, with the nasal mucosa left intact. (6) The nasal layer was sutured. (7) The uvula, palatoglossal arch, and palatopharyngeal arch were reconstructed. (8) A partial tonsillectomy was performed, and finally, the surface layer was sutured (Fig. 6). Postoperatively, the patient had a satisfactory result (Fig. 7).

## Discussion

OSF is a chronic subtle disease linked to significant functional impairment and a higher cancer risk. The exact cause of the disease remains unclear, although it is thought to stem from various factors. Chewing areca nut is the primary cause identified. The major alkaloids found in areca nut, including Arecolidina, Arecoline, Arecaidine, Guacine, and Guyacoline, with Arecoline being the most prevalent, can harm cells and stimulate collagen production [9]. 

A definitive cure for OSF is not yet established. Treatment begins with stopping betel nut chewing, which can lead to regression and symptom improvement in early cases. Various conservative therapies, including oral antioxidants, micronutrients, minerals, turmeric, Pentoxyfilline, Interferongamma, Dexamethasone injections, Hyaluronic acid, Chymotrypsin, Placental extract, milk from immunized cows, stem cell injections, physiotherapy, mouth exercises, and heat therapy, show limited success if started early, and the habit is fully stopped. For severe cases with trismus, dysplasia, or neoplasia, surgical options like Myotomy, Coronoidectomy, and excision of fibrous bands, along with grafts, are recommended [7]. 

Oropharyngeal stenosis and nasopharyngeal stenosis are characterized by the fibrotic narrowing and subsequent fusion of either the anterior pillars, the inferior tonsillar fossa, and the base of the tongue, or the inferior pillars and the posterior pharyngeal wall, respectively. This obliteration of the palatal aperture can lead to a range of symptoms, such as exclusive oral breathing, dysphagia, rhinorrhea, exertional dyspnea, and obstructive sleep apnea [6]. Although rare, oropharyngeal stenosis is primarily observed following adenotonsillectomies. Additionally, it has been recognized as a secondary complication of various diseases, including rhinoscleroma, lupus, diphtheria, tuberculosis, injuries from caustic acids, scarlet fever, Behçet’s disease, and sarcoidosis [8–10]. However, no instances have been reported of it occurring as a secondary complication to OSF. Notably, bilateral buccal OSF caused by chewing betel nut is common, but its potential to cause severe oropharyngeal stenosis has not been documented in previous literature. A possible explanation for the correlation between Betel quid chewing and how it can lead to oropharyngeal stenosis through its role in promoting chronic inflammation and subsequent fibrosis in the oral and pharyngeal tissues. The areca nut, a primary component of betel quid, contains alkaloids like arecoline, which are cytotoxic and contribute to cellular fibrosis and proliferation of fibroblasts. This process can lead to the thickening and scarring of tissues, eventually causing the narrowing of the oropharyngeal cavity, known as stenosis.

The surgical method such as Uvulopalatopharyngoplasty adopted for such cases has achieved a good result. The patient was noncompliant during follow-up, yet upon long-term assessment (8 years postoperatively), a normal mouth opening and oropharyngeal aperture were observed (Fig. 7).

## Conclusion

In conclusion, this case of OSF is reported to be the first incidence of oropharyngeal stenosis. The hypothesis suggests that chewing betel nut leads to progressive scarring, fibrosis, and narrowing of the oropharyngeal aperture. Resection of the fibrotic tissue, with adequate palatoglossal and palatopharyngeal arch reconstruction, is fundamental for success.

## Data Availability

The datasets used and/or analysed during the current study available from the corresponding author on reasonable request.

## Abbreviations

OSF: Oral submucous fibrosis
